# Digital Health for Supporting Precision Medicine in Pediatric Endocrine Disorders: Opportunities for Improved Patient Care

**DOI:** 10.3389/fped.2021.715705

**Published:** 2021-07-29

**Authors:** Luis Fernandez-Luque, Abdullah Al Herbish, Riyad Al Shammari, Jesús Argente, Bassam Bin-Abbas, Asma Deeb, David Dixon, Nabil Zary, Ekaterina Koledova, Martin O. Savage

**Affiliations:** ^1^Adhera Health Inc., Palo Alto, CA, United States; ^2^Dr. Sulaiman Al Habib Medical Group, Riyadh, Saudi Arabia; ^3^National Center for Artificial Intelligence, Saudi Data and Artificial Intelligence Authority, Riyadh, Saudi Arabia; ^4^Department of Pediatrics & Pediatric Endocrinology, Hospital Infantil Universitario Niño Jesús, Instituto de Investigación La Princesa, Universidad Autónoma de Madrid, Madrid, Spain; ^5^Centro de Investigación Biomédica en Red (CIBER) de Fisiopatología de la Obesidad y Nutrición (CIBEROBN), Instituto de Salud Carlos III, Madrid, Spain; ^6^IMDEA Food Institute, CEIUAM+CSIC, Madrid, Spain; ^7^King Faisal Specialist Hospital and Research Centre, Riyadh, Saudi Arabia; ^8^Paediatric Endocrine Division, Sheikh Shakhbout Medical City, Abu Dhabi, United Arab Emirates; ^9^Connected Health and Devices, Merck, Ares Trading SA, Aubonne, Switzerland; ^10^Institute for Excellence in Health Professions Education, Mohammed Bin Rashid University of Medicine and Health Sciences, Dubai, United Arab Emirates; ^11^Global Medical Affairs, Merck Healthcare KGaA, Darmstadt, Germany; ^12^Department of Endocrinology, William Harvey Research Institute, Barts and the London School of Medicine & Dentistry, London, United Kingdom

**Keywords:** digital health, eHealth, artificial intelligence, internet of things, genetics, diabetes mellitus, growth disorders, growth hormone therapy

## Abstract

Digitalization of healthcare delivery is rapidly fostering development of precision medicine. Multiple digital technologies, known as telehealth or eHealth tools, are guiding individualized diagnosis and treatment for patients, and can contribute significantly to the objectives of precision medicine. From a basis of “one-size-fits-all” healthcare, precision medicine provides a paradigm shift to deliver a more nuanced and personalized approach. Genomic medicine utilizing new technologies can provide precision analysis of causative mutations, with personalized understanding of mechanisms and effective therapy. Education is fundamental to the telehealth process, with artificial intelligence (AI) enhancing learning for healthcare professionals and empowering patients to contribute to their care. The Gulf Cooperation Council (GCC) region is rapidly implementing telehealth strategies at all levels and a workshop was convened to discuss aspirations of precision medicine in the context of pediatric endocrinology, including diabetes and growth disorders, with this paper based on those discussions. GCC regional investment in AI, bioinformatics and genomic medicine, is rapidly providing healthcare benefits. However, embracing precision medicine is presenting some major new design, installation and skills challenges. Genomic medicine is enabling precision and personalization of diagnosis and therapy of endocrine conditions. Digital education and communication tools in the field of endocrinology include chatbots, interactive robots and augmented reality. Obesity and diabetes are a major challenge in the GCC region and eHealth tools are increasingly being used for management of care. With regard to growth failure, digital technologies for growth hormone (GH) administration are being shown to enhance adherence and response outcomes. While technical innovations become more affordable with increasing adoption, we should be aware of sustainability, design and implementation costs, training of HCPs and prediction of overall healthcare benefits, which are essential for precision medicine to develop and for its objectives to be achieved.

## Introduction

Precision medicine is not a new principle, but has emerged as a major priority in healthcare delivery, and can be defined as a pathway that employs numerous technologies to guide individually tailored diagnostic methods and treatments for patients (1). The fundamental principle is to personalize medical care to optimize diagnostic efficiency and/or therapeutic benefit by targeting the needs of individual patients on the basis of genetic, biomarker, phenotypic or psychological characteristics (2, 3). Precision medicine incorporates digital health, otherwise known as telehealth or eHealth, which refers to information and communication technologies that are being deployed remotely on a global level. Use of the internet, mobile phones, social media, and computers can bring sophisticated technology to benefit patient care and public health strategies and initiatives. The almost universal use of smart mobile phones allows powerful communication, linked to video technology through dedicated applications (app), directly to the patient or, in the case of pediatrics, to the parent or carer.

The GCC region is rapidly transforming society by the implementation of digitalization strategies at all levels, including the creation of ministries of artificial intelligence (AI) (4–6). In the context of the rapid incorporation of digital health in the GCC region, a workshop was created, with clinical experts from both inside and outside of the region, to discuss how digitalization can foster adoption of precision medicine in healthcare delivery. The discussions primarily considered precision medicine in the context of endocrinology, such as growth disorders, diabetes, and endocrine genetics. The present article was based on the workshop presentations and examines the utilization and aspirations of precision medicine, the challenges for its effective development and implementation, and status in the GCC region.

## Precision Medicine and the Role of Digitalization

Precision medicine is enabling a paradigm shift in the delivery of healthcare from the standardized “one-size-fits-all” strategy to a more nuanced approach. Digital techniques can provide multi-level stratification of patients according to disease sub-types, risk profiles, demographic and socio-economic characteristics, enabling interventions to be delivered on an individual patient level. Factors such as genomics, lifestyle, previous medical history, responsiveness to therapy, and compliance can also be integrated. The aim of precision medicine is to deliver the right intervention to the right patient at the right time.

### The Exposome and Personalization of Healthcare

One of the most challenging elements in precision medicine is the quantification of environmental and lifestyle factors, which is related to the concept of the exposome and affects disease management and therapy outcomes (7, 8). Digital tools can enable capture of data concerning the exposome, based on the interaction of specific external factors, such as individual physical activity and diet, with general factors that influence the internal environment of transcriptomics and proteomics, such as climate, urban vs. rural living and social conditions. The exposome will affect the health risk and have an impact on assessment and management of the individual subject (8–11). The integration of many such individual factors in healthcare delivery presents new challenges. Whereas, care was traditionally calculated on the basis of a cross-sectional snapshot, precision medicine now requires the approach of a longitudinal continuum, because many factors included in the new personalized approach will change during the lifespan of the individual patient.

Precision medicine offers an opportunity for individuals to participate in population research through access to and use of their own genetic data and personal health characteristics, combined with websites such as the 23andme.com app (12–14). Bioinformatic data on growth disorders can take advantage of large cohorts and registries with genetic, biochemical and clinical information, and can use machine-learning to provide predictions of future risks (15–17). Clinical management pathways can be constructed based on tailored diagnosis and treatment algorithms, linking with lifestyle variables, electronic monitoring of responses to therapies and prediction and analysis of health outcomes. Patient feedback can be analyzed, using AI tools, to identify where patient support is needed to change treatment behavior.

Consequently, precision or personalization of healthcare using digital tools is not simply technology, but is a new approach to the practice of medicine. A number of questions exist related to its effective delivery, namely, characterizing variables that need to be personalized, determining effectiveness of precision care compared with standard care, identifying aspects of healthcare most applicable to precision medicine, and ways to best determine the “data points” for personalization.

### Digital Solutions for Medical Education and Outcome Assessment

Medical education may be considered the quickest way to influence healthcare and is multi-factorial, involving health professionals of all levels, healthcare students, and patients and their families. As new digital health devices are made available, patient education becomes highly relevant for increased empowerment of patients to manage their own conditions (18). Education has recently started using AI to enhance learning and support behavioral changes in healthcare management (19).

**Figure 1:**
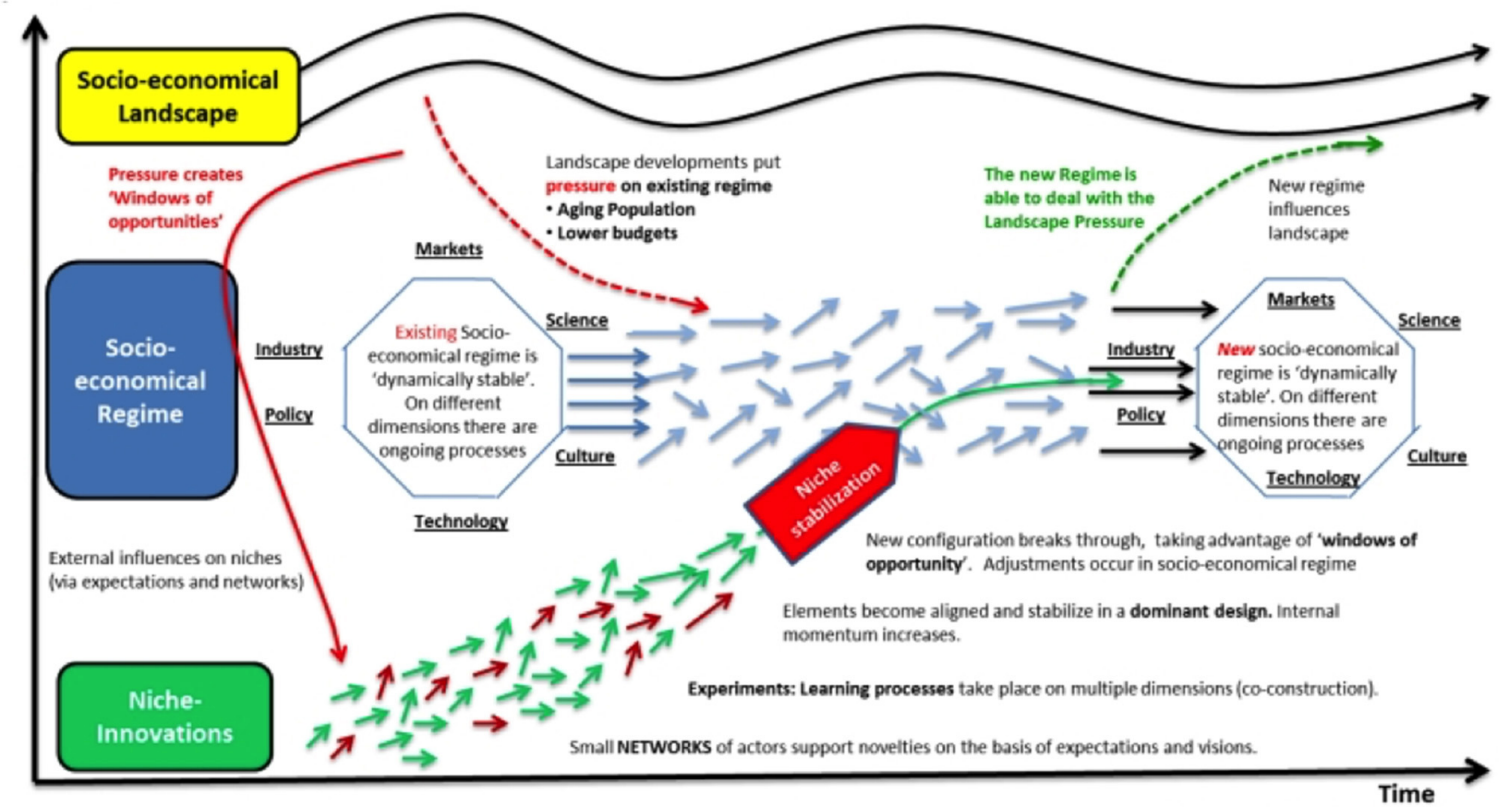
Multi-level perspectives on transitions of the socio-economic regime with increasing structuration of activities in local practices. Small niche innovations in healthcare techniques (shown by short red and green arrows) become organized over time through learning and group support; this feeds into the longer-term socio-economic regime with science, medical practice and healthcare policy forming discrete patterns of technological development (pale blue arrows); the overall socio-economic healthcare landscape changes only very slowly over time (wave-pattern black arrows) and both influences and is influenced by the technological developments of the healthcare socio-economic regime. Adapted with permission from Geels and Schot (20).

Multiple digital tools may be used for collecting data, but it is important to consider what data are of value and decide what can be done with the data to have an impact. This leads to a kind of hidden map where constant niche innovations become aligned over time, while healthcare systems become more complex due to socio-economic factors, aging populations and decreased budgets (20). New ways of working are required, with interactions between niche innovations and socio-economic regimes, creating a new dynamic to local practices and positively influencing the healthcare landscape, as shown in Figure 1. Such changes should, over time, improve the physician-patient relationship.

However, the basic traditions of clinical history-taking and physical examination need to be preserved and cannot be entirely replaced by AI (21). During consultations, it is important that the percentage of time that the clinician is physically engaging with the patient and family is not decreased by pre-occupation with the interpretation of computerized data. While eHealth advances should be welcomed and ideally designed jointly with clinicians and technology experts, there is a risk that the basic principal of quality healthcare, embodied in the doctor-patient relationship, becomes increasingly eroded by new digital advances (22).

## Digital Health Trends in the GCC Region

### The Impact of Healthcare Innovations in the GCC Region

AI and the internet of things (IoT) can provide the world with an abundance of benefits if used optimally, but such developments are presenting new work and skills challenges. Healthcare has developed from descriptive analysis, through diagnostic and predictive analysis, to prescriptive analysis where machine-learning moves to clinical action aimed at providing precision medicine. Within endocrinology, AI models have been designed for diagnosis, prognosis and data analysis, thereby helping to reduce expenses and cope with increasing demands (23); AI has also been used for analyses of genetic data and to indicate gene defects from facial features (23, 24). The challenge is to apply AI and big data to provide beneficial clinical insights from the heterogeneous information (23, 25).

The IoT is being used for multiple healthcare purposes, such as glucose monitoring and diagnosis of cardiovascular disease (26, 27). In Saudi Arabia, the IoT and big data are being developed to detect and assess patients with chronic conditions, to minimize disease risks (28). In the next 10 years, the potential impact of AI in the Middle East is anticipated to be US$320 billion, 11% of the overall GDP (29). AI is expected to provide 12.4% of the gross domestic product (GDP) of the Saudi Arabia economy and almost 14% of the GDP of the UAE (6); in the Middle East about 19% of GDP will come from the health and education sector (29).

In the GCC region, life expectancy has increased and infant mortality has decreased, resulting in an increasing population (4, 30). The population of the GCC countries is about 55 million and more than half are under the age of 40 years (31). In 2018, about 92% used the internet and about 45 million used social media, with mobile phone use higher than the global average (31). However, there is a major challenge of obesity and diabetes mellitus in the region (32), with concerns raised with political leaders, and there have been various initiatives in individual countries; there is an expectation that technology will make the region's health services more effective (28, 30, 33).

### Precision Medicine in the GCC Region

Current models for healthcare delivery are an amalgam from various countries and cultures, such as the US, Europe, Africa, Southeast Asia and Middle East. The GCC region resembles Southeast Asia to some extent, with some European and North American influences, but clinics need to create their own models. The GCC countries have acknowledged that digitalization is important to improve patient care, but lack of digitalized data and training of health care professionals (HCP) are barriers to current use of precision medicine (6, 34). GCC countries are increasingly investing in bioinformatics and genomic medicine, enabled by next generation sequencing, the IoT and big data. In the context of growth disorders, genomic information provides accuracy of causative mutations, personalized understanding of disease mechanisms, and effective therapies. However, challenges remain in the integration of such data into clinical practice.

There is a need for collaboration between technology experts, physicians and citizens (25), which is especially challenging in a region with a significant reliance on a foreign healthcare workforce and the related training issues (4, 6). The existence of electronic health records is generally seen as a positive development to aid adoption of precision medicine, with barriers informing changes in strategy (35). Telemedicine, involving remote delivery of care, is becoming more common in the GCC region, with new initiatives such as the Saudi Telemedicine Unit of Excellence now established (36, 37). The accuracy and safety of collected data and ethical approval for its use are additional factors. Also, while increasing implementation of technical innovations generally makes them more affordable, there remain significant implementation and sustainability costs of digitalization and precision medicine for national medical care systems (4).

Health tourism has become a recent issue in the region. Some countries focus on clinical quality and others on promotion of health tourism, and there needs to be a balance of these values. A new system has been designed in the United Arab Emirates (UAE) to view summaries of clinical information analyzed from individual healthcare files, allowing HCPs to choose appropriate management plans for each patient (4, 38). The summary contains full patient details, including electronic growth charts, and is used to assess diagnosis and progress, with abnormal results highlighted for attention. Patients have access to their data and can create reminders and organize communication with the HCPs. A mobile app in the system integrates with other devices, such as glucometers, blood pressure monitors, and weighing scales. Thus, GCC countries have made good progress at both government and private levels, with benefits for many diseases including pediatric endocrine diseases.

## Genomics in Precision Medicine

### Integrating Genetics as a Precision Tool Into Clinical Practice and Clinical Support Systems

The rapidly advancing field of genomic medicine is providing new knowledge related to precision, personalization, prevention, and participation for diagnosis and therapy. It enables physicians to be certain of the diagnosis, aiming to know and understand the pathogenesis of a disorder to enable consideration of the best therapy, and technologies, including genotyping, functional studies and gene-altered animal models, can directly contribute to the aims of precision medicine. Following DNA sequencing, variants can be classified as pathogenic, likely pathogenic, of uncertain significance, benign or likely benign; interpretation of these results depends on supporting evidence from clinical and biochemical data (17, 39). The field of pharmacogenomics, which involves the effects of genetic variants on drug metabolism and, therefore, the responsiveness of individuals to specific therapies, is also directly relevant to precision medicine (40–43).

Next generation sequencing is making an enormous difference in clinical diagnosis compared to conventional techniques of Sanger or candidate gene sequencing (44, 45). New techniques in genetic studies can be applied to single genes, multiple gene panels, whole exome and genome sequencing, and analysis of possible transcriptome and epigenetic abnormalities. Current challenges facing geneticists include decisions on type of DNA sequencing appropriate for each clinical situation, and best choice of laboratory and facilities for data interpretation. Patient concerns of confidentiality and control over genomic data must also be investigated and addressed for integration into healthcare (46, 47). Examples of genetic analysis linked to precision medicine are seen in the investigation of the multiple causes of short stature, including epigenetic defects, syndromic growth failure and the search for causative genetic variants of obesity of endocrine—either pro-opiomelanocortin (POMC) deficiency or leptin receptor (LEPR) deficiency obesity—or syndromic origin (17, 41, 45, 48–52).

### Precision of Single Gene Defects in Clinical Disorders of Linear Growth

Laron syndrome, caused by autosomal recessive inheritance of a homozygous or compound heterozygous mutation of the GH receptor gene (*GHR*), is a severe form of GH resistance and presents a classical paradigm of congenital insulin-like growth factor (IGF)-1 deficiency (53, 54). The mutation translates into a functionally inactive GHR protein, which disturbs IGF-1 production, severely interfering with post-natal linear growth. Mutation variants have been reported in exons coding for the extracellular, transmembrane and intracellular domains of the protein, leading to subtle differences in the phenotype and degree of short stature (55, 56). Further study of populations of adult patients with Laron syndrome has revealed that living with severe and chronic IGF-1 deficiency carries protection against diabetes mellitus, cardiovascular and malignant diseases, and an advantage in terms of longevity (57, 58).

Another example of precision genetic investigation is the discovery of autosomal recessive homozygous loss-of-function mutations in the gene for pregnancy-associated plasma protein A2 (*PAPPA2*, pappalysin2), identified from whole exome sequencing (59). The PAPP-A2 protein is an enzyme that cleaves IGF binding protein (IGFBP)-3 and IGFBP-5 within the circulating ternary complex, thereby releasing free IGF-1 for its physiological effects (59, 60). Patients with mutations of this enzyme have very low levels of bioactive free IGF-1, associated with poor growth (39, 61). *PAPPA2* mutations represent a good example of how genetic investigation has been able to pinpoint a new physiological process that, if disturbed, translates into an abnormal clinical phenotype associated with diagnostic biochemical features (17, 62). Understanding of this abnormality points to therapeutic intervention using recombinant human IGF-1, which has been shown to be effective in stimulating growth in affected subjects (63). Consequently, the precision medicine goals of accuracy of pathogenesis, personalization of the mutation, and indication of a targeted therapeutic intervention are all achieved by the PAPP-A2 deficiency syndrome.

Clinical disorders of growth have highly complex pathogenetic origins, with broad phenotypic variability shown by proportionate or disproportionate and mild to severe short stature. The origin may be pre-natal or post-natal and the primary defect may involve endocrine function or growth plate physiology (17). Genetic investigations embrace both pragmatic and research-based activities and new genetic associations are being identified as new technologies become more sensitive, cheaper, and widely applied. Novel genes and genetic pathways are being identified that affect pituitary function and are associated with post-natal growth failure, such as bone morphogenic protein (*BMP*) gene mutations (64), and the multiple genes involved in RASopathies, including some, such as *LZTR1* mutations, that correlate with the Noonan syndrome phenotype (65, 66). Many of these short stature genetic variants respond to GH therapy, although not all (17, 64, 67). Hence, clinicians should be continuously up-dated of new developments and actively encouraged to build working relationships with genetic laboratories to discuss the indications and potential treatment options (17).

## Applying Digital Technologies to Advance Long-Term Care of Endocrine and Metabolic Disorders

Digital tools such as video games, have been used for education in children with diabetes mellitus for more than 20 years (68–70), and patients with conditions including irritable bowel disease, cystic fibrosis, and type 1 diabetes have shown improvements with online support during transition from pediatric to adult care (71). New channels for education and communication are being developed, such as chat-bots, interactive robots, and augmented reality for patients with diabetes (72–74). Chatbots are AI-based semi-automatic conversational agents that have grown in importance for behavioral change interventions (72, 75). Personalized robots for diabetes education and daily management have become increasingly affordable and accepted (76). Also, the increased power of mobile phones is allowing the creation of apps that use the camera to allow children to “augment” the reality by adding additional information, such as identifying the carbohydrate content of foods (74) and demonstration of correct injection techniques (77). Wearable devices with cameras are being developed that can be used to assess dietary intake (78), and can interact with deep-learning computer techniques for sleep quality evaluation (79–81). In patients with diabetes, digital devices and machine-learning have been used to assess management (82), and predict risk of heart failure (83) or nephropathy (84). However, care must be taken to assess specific challenges. Studies of devices to influence lifestyle of obese children found that data collection was affected by gender aspects of patients and parents (85), and physical activity patterns differ between countries (86). Therefore, global benchmarking is needed for AI tools in medicine (87).

In growth disorders, continuous monitoring, and support programs are needed to enable good long-term adherence. These are required to overcome factors such as patients' lack of information, limited knowledge of the condition and treatment effects, socio-economic aspects, and health system variabilities. Thus, adherence strategies need to be focused and personalized, with behavioral aspects linked with clinical outcomes (88). Questions remain regarding what digital health tools are needed by clinicians and patients; they should be part of integrated care, with all stakeholders involved in design (89). This is important to ensure the utility and usability of the systems, and also to ensure the safety and confidentiality of digital health information and electronic health records. There are concerns about the quality of mobile health apps that might contain misleading information (90), and privacy risks have been widely reported in apps in diverse therapeutic areas (91–93). With security risks continuously evolving, a key aspect to consider is the constant need for updating the training of clinicians, caregivers, and patients to maximize their digital health literacy skills for technologies to be used safely (94, 95).

### Digital Technologies in the Care of Patients With Diabetes Mellitus

Pediatric obesity and diabetes is becoming an increasing problem in Saudi Arabia (32, 96). The majority of children with type 1 diabetes have suboptimal glycemic control, increasing their risk of complications. Technology to support diabetes self-care has advanced significantly, including insulin pump therapy, continuous glucose monitoring, and sensor-augmented pump therapy (97–99), which are stepping stones toward the “artificial pancreas” using closed-loop technology. Local studies in Saudi children with diabetes showed that continuous subcutaneous insulin infusion improved glycemic control, with decreased hypoglycemic episodes and diabetic ketoacidosis events (100, 101). The widespread use of mobile phones offers opportunities for continuous digital glucose monitoring, with message alerts and sharing of data with healthcare providers (102), and the feasibility of remote monitoring of insulin delivery should be addressed among children with diabetes and their caregivers.

Digital apps can directly support patients and their carers to improve diabetes care and self-management (103, 104). Educational support using short messaging services or health apps was associated with improved glycemic control in both type 1 and type 2 diabetes (105, 106). In a study of 200 children with type 1 diabetes, 5–7 text messages per week, aimed at increasing diabetes knowledge and education, were sent to patients and/or parents for 6 months. Messages included video clips, such as on injection technique and glucometer usage. Significant improvements were seen for glycemic control, frequency of monitoring, number of missed injections, and mean score in parent's knowledge test (105).

A similar study in patients with type 2 diabetes showed less dramatic results, but there were still improvements in fasting blood glucose, glycated hemoglobin, and patients' knowledge (106). Following on from these results, new mobile phone apps are being developed in GCC countries, including diagnostic testing and gamification (107, 108). However, there remain problems of funding by individual healthcare authorities and difficulties of acceptance of self-management by patients, and cultural differences mean that interventions should be regionally appropriate (18, 108).

### Digital Technologies for Adherence to GH Therapy

Chronic disorders that require continuous therapy over long periods of time inevitably face problems of maintaining adherence, and digital technologies are being used to assist with this (109, 110). Electronic monitoring of GH injections has been a major advance in improving adherence to pediatric GH treatment regimens (111, 112). The easypod™ auto-injector uses the web-based easypod connect platform that allows adherence data to be transmitted electronically to healthcare clinicians, who can then easily analyze GH treatment history, enhancing real-world healthcare decisions. The easypod connect system may be considered as an integrated electronic communication system rather than simply an injection device. Because it is computer-based, multiple information can be entered and stored, which can include outcomes such as height and weight, enabling growth to be assessed relative to other patients. Adherence can be viewed in different formats according to physician preference, and features such as comfort and dosage settings can be easily seen and adjusted, and may help to avoid wastage.

The multi-national easypod connect observational study (ECOS) assessed adherence among pediatric patients using the easypod connect system for GH therapy for up to 5 years. Adherence was ≥80% for more than 3 years for results overall and for several individual countries, and analyses showed significant associations between poor adherence and impaired clinical outcomes (112–117). Adherence rates for patients new to GH therapy and those already receiving GH did not differ, and there was no difference between diagnoses (112). Further analyses using AI to identify types of patients with reduced adherence have indicated worse adherence relating to factors such as being male, puberty and starting to self-inject, and that young children have better adherence with daily injections while older patients, who have used easypod for more than 12 months, have better adherence with a 6-day injection regimen.

Results from 56 patients in ECOS in the UAE showed adherence within the first month was ≥85% for 78% of patients, but fell to 47% for 15 patients with 12-month data. The initial data were very similar to those seen in other countries, such as in the Middle East and Asia, and further analysis is required to understand why this adherence pattern occurs. For 53 patients treated with the easypod device at Mafraq Hospital, UAE, 39 (74%) were male and age range at start was 5–15 years, median 8.2 years. The indication for GH therapy was mainly GH deficiency (53%), followed by small for gestational age (19%) and idiopathic short stature (15%). A utilization and satisfaction questionnaire completed by 32 of these patients indicated that a majority considered the skin sensor, pre-dose features, and indicator of battery charge as useful or very useful. However, many patients and parents were not aware of tracking features in the device, suggesting that improved training is required.

Such information from the easypod connect system provides a database for auditing and research. Patient questionnaire feedback can be helpful to improve injection technique and optimize adherence, and advanced functions may help alleviate complications faced by patients. The UAE data are being further analyzed to examine factors influencing adherence, such as socio-economic characteristics, which will enable reduced adherence to be addressed through patient support programs and eHealth tools to enhance behavioral changes.

### Self-Reported Growth Information and Patient Education

Self-reported outcomes of GH therapy represent a new electronic challenge. While height is recorded on patient health records <1% of clinics worldwide currently record height data in the easypod connect platform. At present, there are no clinically-validated consumer height measurement tools available; however, ways of capturing height data with the easypod are being examined. A promising technique uses augmented reality for measuring vertical surfaces, which is now available on many mobile devices and has already been used in medicine for visualizing human anatomy (118–121). To the best of our knowledge, there has been no clinical validation of such methods in routine clinical practice and patients' homes. This is important because validity of measurement can greatly depend on how the digital technique is being applied. Studies have been not only limited to measuring height, but also body fat composition using medical images as gold standard for comparison (122).

The augmented reality application is also being investigated for training of patients and caregivers in the use of the easypod device (123). The system aims to empower patients when starting easypod use, and reduce the burden on physicians and nurses by helping patients to understand device settings and choose which options to use.

The growlink smartphone app, launched in 2019, provides information to patients and caregivers to enable them to see the GH treatment history, similar to the connect platform, and provides injection reminders (124). However, growlink also enables patients to self-report their height and weight data, which is transmitted to HCPs to facilitate growth tracking. The app also allows patients to see their growth records, and access educational materials on GH requirements and use of the easypod injection device. This helps patients become engaged in their treatment and have more meaningful discussions with their HCPs.

## Conclusions

For precision medicine and technological innovations to be integrated into routine clinical practice, input is required from many sources. There are no simple solutions to this process. Collaborations between technology designers and clinical physicians are essential to assess the clinical needs and to identify the questions that innovations can begin to answer. Expert economic assessment of the investment required for such technology needs to be balanced against the economic gains of its installation, implementation, and use. Training of clinicians is essential and must be factored into the overall costs. The unmet clinical needs should be clearly identified, both in the fields of public health and specialist care. Implementation research is needed, with progress in digital health made in small steps rather than large over-ambitious steps.

## Author Contributions

The above article reflects presentations and discussions that took place at a workshop, ELITE V. All authors contributed to presentations and discussions at the workshop and have made a substantial, direct and intellectual contribution to the article, and have approved it for publication.

## Conflict of Interest

LF-L is an employee of Adhera Health Inc., Palo Alto, CA, United States. AA has served as a local principal investigator for the ECOS study conducted by Merk Serono Middle East FZ-LLC. DD is an employee of Ares Trading SA, Aubonne, Switzerland. EK is an employee of Merck KGaA, Germany. MS has acted as a consultant to Merck KGaA, Germany, Sandoz, OPKO and Genexine. The remaining authors declare that the research was conducted in the absence of any commercial or financial relationships that could be construed as a potential conflict of interest.

## Publisher's Note

All claims expressed in this article are solely those of the authors and do not necessarily represent those of their affiliated organizations, or those of the publisher, the editors and the reviewers. Any product that may be evaluated in this article, or claim that may be made by its manufacturer, is not guaranteed or endorsed by the publisher.
